# First-Principle Studies on the Mechanical and Electronic Properties of Al_x_Ni_y_Zr_z_ (x = 1~3, y = 1~2, z = 1~6) Alloy under Pressure

**DOI:** 10.3390/ma13214972

**Published:** 2020-11-05

**Authors:** Xiaoli Yuan, Weikang Li, Peng Wan, Mi-An Xue

**Affiliations:** 1 College of Science, Hohai University, Nanjing 210024, China; liweikang21@126.com (W.L.); sunsetglow3@hhu.edu.cn (P.W.); 2 Key Laboratory of Ministry of Education for Coastal Disaster and Protection, Hohai University, Nanjing 210024, China

**Keywords:** Al_x_Ni_y_Zr_z_, first-principle, electronic, mechanical properties, high pressure

## Abstract

The elastic and electronic properties of Al_x_Ni_y_Zr_z_ (AlNiZr, Al_2_NiZr_6_, AlNi_2_Zr, and Al_5_Ni_2_Zr) under pressure from 0 to 50 GPa have been investigated by using the density function theory (DFT) within the generalized gradient approximation (GGA). The elastic constants *C_ij_* (GPa), Shear modulus *G* (GPa), Bulk modulus *B* (GPa), Poisson’s ratio *σ*, Young’s modulus *E* (GPa), and the ratio of *G/B* have been studied under a pressure scale to 50 GPa. The relationship between Young’s modulus of Al_x_Ni_y_Zr_z_ is Al_5_Ni_2_Zr > AlNiZr > Al_2_NiZr_6_ > AlNi_2_Zr, which indicates that the relationship between the stiffness of Al_x_Ni_y_Zr_z_ is Al_5_Ni_2_Zr > AlNiZr > Al_2_NiZr_6_ > AlNi_2_Zr. The conditions are met at 30 and 50 GPa, respectively. What is more, the *G/B* ratios for AlNiZr, AlNi_2_Zr, Al_2_NiZr_6_, and Al_5_Ni_2_Zr classify these materials as brittle under zero pressure, while with the increasing of the pressure the *G/B* ratios of AlNiZr, AlNi_2_Zr, Al_2_NiZr_6_, and Al_5_Ni_2_Zr all become lower, which indicates that the pressure could enhance the brittle properties of these materials. Poisson’s ratio studies show that AlNiZr, AlNi_2_Zr, and Al_2_NiZr_6_ are all a central force, while Al_5_Ni_2_Zr is a non-central force pressure scale to 50 GPa. The energy band structure indicates that they are all metal. The relationship between the electrical conductivity of Al_x_Ni_y_Zr_z_ is Al_2_NiZr_6_ > Al_5_Ni_2_Zr > AlNi_2_Zr > AlNiZr. What is more, compared with Al_5_Ni_2_Zr, AlNi_2_Zr has a smaller electron effective mass and larger atom delocalization. By exploring the elastic and electronic properties, they are all used as a superconducting material. However, Al_5_Ni_2_Zr is the best of them when used as a superconducting material.

## 1. Introduction

Ni-Al intermetallics are widely used as a novel high temperature structural material and aerospace material due to their high melting point, good oxidation resistance, and thermal conductivity. Shi et al. [1] by using the first-principle studied the electronic properties, elastic properties, structural properties, and the formation of Al-Ni intermetallics compounds. Wang et al. [2] studied the thermodynamic of Ni_3_Al from the first-principle calculation. Chao et al. [3] by taking the first-principles, plane-wave method in combination with ultra-soft, pseudopotentials predict the crystal structures, lattice parameters, volumes, elastic constants, bulk moduli, and shear moduli of the binary NiAl. Yu et al. [4] investigated the self-diffusion in NiAl and Ni_3_Al by the molecular dynamics (MD) with an analytical embedded atom and resolved this problem by incorporating Zr into Al-Ni to form the ternary Al-Ni-Zr [5,6].

However, its low ductility limited its application. It is an effectual way to enhance the oxidation resistance properties of the Ni-Al intermetallics by incorporating Zr into Al-Ni to form the ternary Al-Ni-Zr [7,8], which arouses people’s interest in calculating the Zr-droped Ni-Al intermetallics theoretically [9,10,11] and experimentally [12,13,14,15]. Wu et al. [16] studied the lattice misfit on the occupational behaviour and ductility properties with others, and found in energy analysis that the preferable site of Zr between Ni sublattice and Al sublattice will change under a different lattice misfit. Yang et al. [17] calculated the Gibbs energy of Zr-Al-Ni based on the quasi-regular solution model. Li et al. [18] conducted the research on the solid-liquid interfacial energy for Al-Ni-Zr alloys. However, there are limited reports on the elastic, structure, and electronic properties of Al-Ni-Zr intermetallics, including tetragonal Al_5_NiZr_2_, hexagonal Al_2_NiZr_6_, and AlNiZr, as well as the cubic AlNi_2_Zr under pressure. What is more, the pressure dependence of the band gap and elastic properties for Al_x_Ni_y_Zr_z_ is important to understand the effect of strain on the alloys and many practical applications [19]. Studying their properties can help us explore their potential and application value in the aerospace field. The first-principle calculation with the pseudopotential method based on DFT has grown up to be a standard tool for the calculation of the material modeling simulation [20,21,22]. Therefore, in this paper, the first-principle calculation with DFT and GGA is utilized to calculate the elastic and electronic properties.

## 2. Computational Method and Theory

The numerical calculations are done by using DFT with GGA and is performed by the exchange-correlation energy in the scheme of the Perdew-Burke-Ernzerhof (PBE). The pseudo atoms calculated are Al (3s^2^ 3p^1^), Ni (3d^8^ 4s^2^), and Zr (4s^2^ 4p^6^ 4d^2^ 5s^2^). In order to guarantee the accuracy of the calculation results, after repeated testing, we chose the cut-off energy of 600 eV for the wave function and charge density expansion. The k-point meshes of 4 × 4 × 8 for AlNiZr and Al_2_NiZr_6_, 6 × 6 × 6 for AlNi_2_Zr, as well as 14 × 14 × 14 for Al_5_Ni_2_Zr, respectively were used to model the first Brillouin zone. The elastic properties were investigated by using the optimized stable structure. All calculations were done through the quantum mechanics software CASTEP [23].

AlNiZr and Al_2_NiZr_6_ belong to the hexagonal crystal system with space group P6-2m and they have five independent components *C*_11_, *C*_33_, *C*_44_, *C*_12_, and *C*_13_, their stability equation was derived from [24]. AlNi_2_Zr belongs to the cubic system with space group FM3-M and its elastic tensors *C_ij_* have three independent components *C*_11_, *C*_12_, and *C*_44_, whose equations are derived from [25]. Al_5_Ni_2_Zr with space group 14/MMM belongs to the tetragonal system and it has five independent components *C*_11_, *C*_12_, *C*_13_, *C*_33_, *C*_44_, and *C*_66_, whose stability equations are derived from [24]. The Voigt band and the Reuss band show the upper band and the lower band, respectively. Additionally, the Voigt-Reuss-Hill approximation means the arithmetic of the two bands [26]. *B* and *G* express the Bulk modulus and Shear modulus, respectively. *V*, *R*, and *H* indicate the Voigt band, Reuss band, and Hill average, respectively. They are generated from the following equations.

For the cubic system [27]:

(1) $$B_{V}=B_{R}=\frac{C_{11}+2C_{12}}{3}$$

(2) $$G_{V}=\frac{C_{11}-C_{12}+3C_{44}}{5}$$

(3) $$G_{R}=\frac{5C_{44}(C_{11}-C_{12})}{4C_{44}+(C_{11}-C_{12})}$$

The criterion of mechanical stability is [28,29,30], *P* is the pressure.

$$C_{11}+2C_{12}+P>0,C_{44}-P>0,C_{11}-C_{12}-2P>0$$

For the tetragonal system [31]:

(4) $$B_{R}={\left[(2S_{11}+S_{33})+2(S_{12}+2S_{13})\right]}^{-1}$$

(5) $$B_{V}=\frac{1}{9}(2C_{11}+C_{33})+\frac{2}{9}(C_{12}+2C_{13})$$

(6) $$G_{R}=15{\left[4(2S_{11}+S_{33})-4(S_{12}+2S_{13})+3(2S_{44}+S_{66})\right]}^{-1}$$

(7) $$G_{V}=\frac{1}{15}(2C_{11}+C_{33}-C_{12}-2C_{13})+\frac{1}{5}(2C_{44}+C_{66})$$

*S_ij_* is the inverse matrix of the elastic constants matrix *C_ij_*.

The criterion of mechanical stability is [32]:

$$\begin{array}{l} (C_{ii}-P)>0(i=1,2,\ldots 6);(C_{11}-C_{12}-2P)>0;\\ (C_{11}+C_{33}-2C_{13}-4P)>0 \end{array}$$

For the hexagonal system [33]:

(8) $$B_{R}=\frac{(C_{11}+C_{12})C_{33}-2{C_{13}}^{2}}{C_{11}+C_{12}+2C_{33}-4C_{13}}$$

(9) $$B_{V}=\frac{1}{9}[2(C_{11}+C_{12})+C_{33}-4C_{13}]$$

(10) $$G_{R}=\frac{5}{2}\frac{{\left((C_{11}+C_{12})C_{33}-2{C_{13}}^{2}\right)}^{2}C_{44}C_{66}}{3B_{V}C_{44}C_{66}+{\left((C_{11}+C_{12})C_{33}-2{C_{13}}^{2}\right)}^{2}(C_{44}+C_{66})}$$

(11) $$G_{V}=\frac{1}{30}(C_{11}+C_{12}+2C_{33}-4C_{13}+12C_{44}+12C_{66})$$

The criterion of mechanical stability is [29]:

$$C_{44}-P>0;(C_{11}-P)>\left|C_{12}+P\right|;(C_{33}-P)(C_{11}-P+C_{12}+P)>2(C_{13}+P)$$

The *B_R_*, *B_V_*, *G_R_*, and *G_V_* could be obtained by the calculation of Equations (1)–(11).

The average values under the Voigt and Reuss bounds can be expressed by the modulus of polycrystal, while under the Voigt-Reuss-Hill approximation [30,32,34]:

(12) $$\begin{array}{l} B=\frac{1}{2}(B_{V}+B_{R}),\\ G=\frac{1}{2}(G_{V}+G_{R}), \end{array}$$

Young’s modulus (*E*) and Poisson’s ratio (*σ*) were obtained by these equations:

(13) $$\begin{array}{l} E=\frac{9BG}{3B+G},\\ \sigma =\frac{3B-2G}{2(3B+G)}. \end{array}$$

## 3. Results and Discussion

### 3.1. The Elastic Properties under Pressure

**Figure 1:**
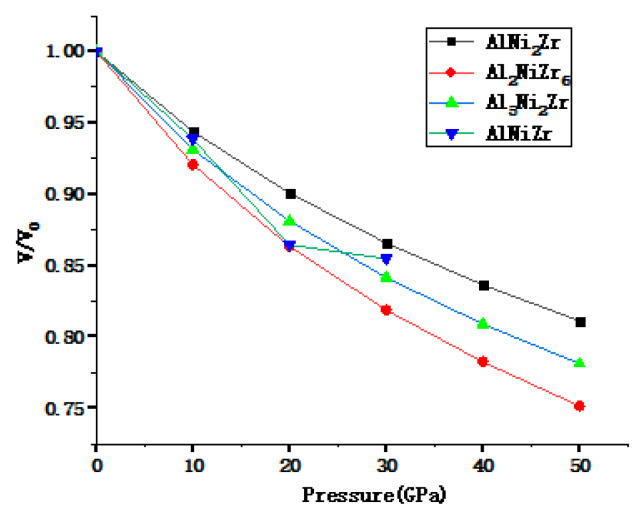
Calculated pressure dependence of the volume for Al_x_Ni_y_Zr_z_.

To further understand the influence of the external pressure on the lattice parameters of Al_x_Ni_y_Zr_z_ alloys, the relative change equilibrium volume of Al_x_Ni_y_Zr_z_ in the range of 0–50 GPa is optimized and calculated with a step of 10 GPa, and the pressure and volume curves are drawn in Figure 1. The relative volume V/V_0_ decreases with the increasing of the pressure, as shown in Figure 1.

**Figure 2:**
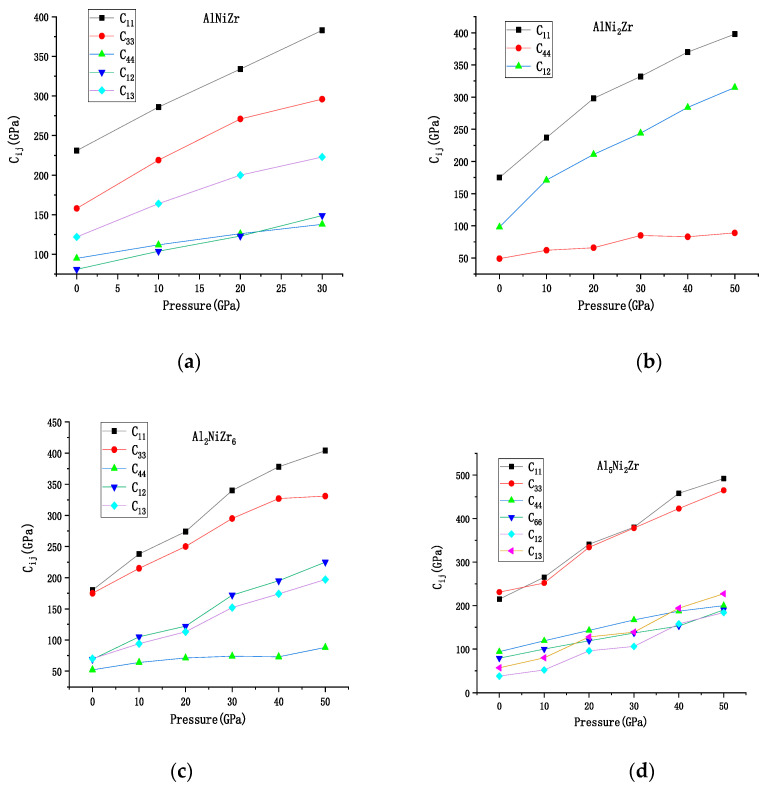
Calculated pressure dependence of the elastic constants C_ij_ for Al_x_Ni_y_Zr_z_ compounds: (**a**) AlNiZr, (**b**) AlNi_2_Zr, (**c**) Al_2_NiZr_6_, (**d**) Al_5_Ni_2_Zr.

The elastic constants were calculated using a linear fit of the stress-strain function. The relations calculated the elastic constant *C_ij_* of Al_x_Ni_y_Zr_z_ with the pressure, as shown in Figure 2. The *G* (GPa), *B* (GPa), *σ*, *E* (GPa), and the ratio of *G/B* at various pressures are listed in Table 1. Unfortunately, there are no experimental and theoretical elastic parameters to compare with Al_x_Ni_y_Zr_z_. First of all, the elastic constant of AlNiZr only meets the hexagonal mechanical stability criteria when *P* < 30 GPa. When the pressure ranges from 0 to 50 GPa, the Al_2_NiZr_6_, AlNi_2_Zr, and Al_5_Ni_2_Zr are of mechanical stability and with no phase transformation until the pressure is up to 50 GPa. Therefore, in this paper, the elastic properties of Al_2_NiZr_6_, AlNi_2_Zr, and Al_5_Ni_2_Zr were studied and only the elastic and structural properties of AlNiZr under 30 GPa were studied. The data indicated that the relationship between Young’s moduli of Al_x_Ni_y_Zr_z_ is Al_5_Ni_2_Zr > AlNiZr > Al_2_NiZr_6_ > AlNi_2_Zr, which indicated that the relationship between the stiffness of Al_x_Ni_y_Zr_z_ is Al_5_Ni_2_Zr > AlNiZr > Al_2_NiZr_6_ > AlNi_2_Zr. The conditions are satisfied at 30 and 50 GPa, respectively.

The variation relation of the elastic constants of Al_x_Ni_y_Zr_z_ compounds with the pressure is shown in Figure 2. It can be learned from Figure 2 that a linear dependence in all the curves of these compounds are in the considered range of pressure. For Al_2_NiZr_6_, AlNi_2_Zr, and AlNiZr, with the change of pressure *C*_11_ changes the most compared with the other elastic moduli. *C*_44_ changes the least with the pressure. As for Al_5_Ni_2_Zr, *C*_11_ and *C*_33_ are more sensitive to the change of pressure compared with *C*_44_, *C*_66_, *C*_12_, and *C*_13_.

From the relevant literature, it can be learned that G represents the plastic deformation resistance of the material and B represents the fracture resistance of the material [35]. Pugh proposed that the *G/B* ratio is used to estimate the toughness of the corresponding materials [36]. According to his theory, a small *G/B* value indicates the toughness of the corresponding material, while a larger *G/B* value corresponds to brittleness. The critical separation value of ductile and brittle materials is around 1.75. If *G/B* > 1.75, the material is ductile, otherwise, the material is brittle [37].

**Figure 3:**
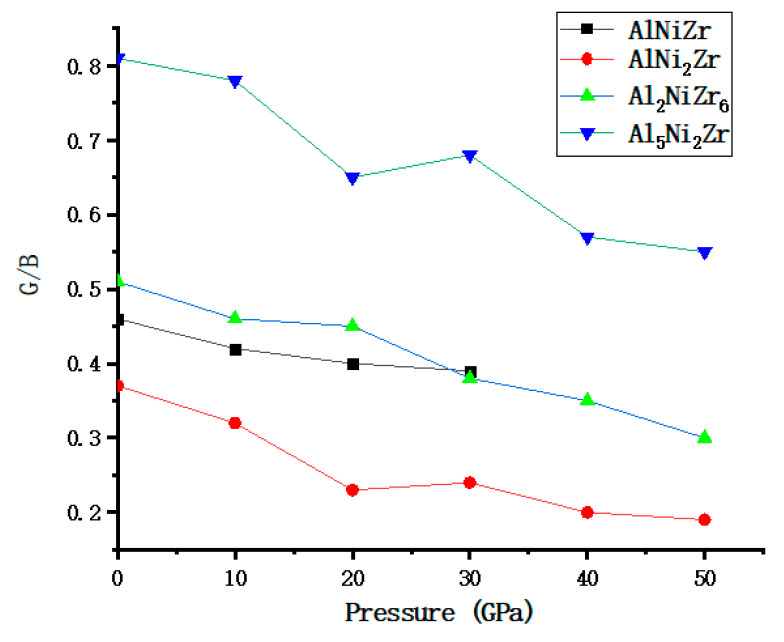
The dependence relations of *G/B* values with pressures for Al_x_Ni_y_Zr_z_ compounds.

The relations of Al_x_Ni_y_Zr_z_ with the pressure and for AlNiZr, AlNi_2_Zr, Al_2_NiZr_6_, and Al_5_Ni_2_Zr are presented in Figure 3 and Table 1. The *G/B* ratios are respectively 0.457, 0.366, 0.372, 0.509, and 0.813 under zero pressure, classifying these materials as brittle. However, with the increasing of the pressure, the *G/B* ratios of AlNiZr, AlNi_2_Zr, Al_2_NiZr_6_, and Al_5_Ni_2_Zr all become lower which indicate that for these materials the pressure could enhance the brittle properties of the materials. The binding force of the atom can be shown by Poisson’s ratios. Poisson’s ratio of covalent materials is small (0.1), while the typical value of ionic materials is 0.25 [38]. Therefore, the ionic contribution of these compounds to the interatomic bond is dominant. The value of Poisson’s ratio represents the degree of directionality of covalent bonds. The values of 0.25 and 0.5 are the lower and upper limits of the central force solid, respectively [39]. As shown in Table 1, Poisson’s ratios of AlNiZr, AlNi_2_Zr, and Al_2_NiZr_6_ are both bigger than 0.25 under the pressure from 0 to 50 GPa, similar to the AlNiZr under the pressure from 0 to 30 GPa, which shows that they are a central force. However, Poisson’s ratios of Al_5_Ni_2_Zr are less than 0.25 under the pressure from 0 to 30 GPa, which shows that it is a non-central force, while it is a central force when the pressure is higher than 30 GPa.

### 3.2. The Electronic Properties

**Figure 4:**
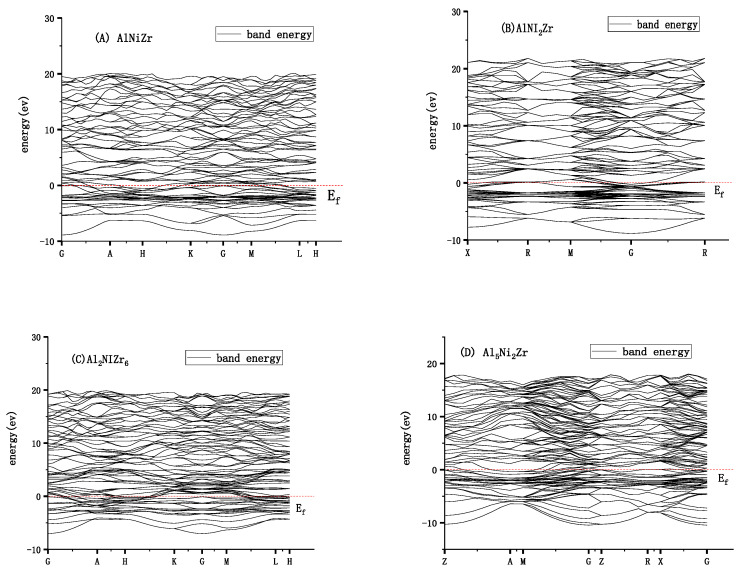
Band structures of Al_x_Ni_y_Zr_z__,_ (**A**) AlNiZr, (**B**) AlNi_2_Zr, (**C**) Al_2_NiZr_6_, and (**D**) Al_5_Ni_2_Zr.

The calculated energy band structure of Al_x_Ni_y_Zr_z_, (A) AlNiZr, (B) AlNi_2_Zr, (C) Al_2_NiZr_6_, and (D) Al_5_Ni_2_Zr along with the high-symmetry directions in the Brillouin zone is shown in Figure 4.

**Figure 5:**
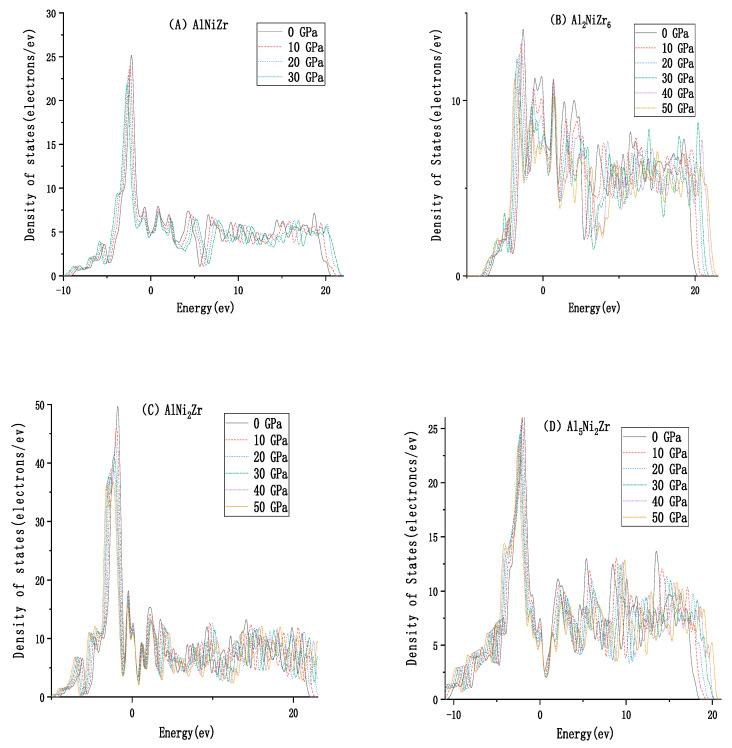
Total density of states versus pressure of (**A**) AlNiZr, (**B**) Al_2_NiZr_6_, (**C**) AlNi_2_Zr, (**D**) Al_5_Ni_2_Zr.

The Fermi level is the highest level at absolute zero. According to Pauli’s exclusion principle, a quantum state cannot hold two or more fermions. At absolute zero degrees, the electrons will fill the energy levels successively from low to high, except for the highest energy level, which will form the Fermi sea of electronic state. The plane of the Fermi sea is the Fermi level. The prerequisite for a good conductor is the case that the Fermi level intersects one or more energy bands. As shown in Figure 4, all the energy bands of Al_x_Ni_y_Zr_z_ pass through the Fermi surface, indicating that they are conductors. For AlNiZr and Al_2_NiZr_6_ with the hexagonal system, the electrical conductivity of Al_2_NiZr_6_ is preferable to AlNiZr, due to more energy bands that pass through the Fermi surface for Al_2_NiZr_6_. For AlNi_2_Zr and Al_5_Ni_2_Zr with the cubic system, the electrical conductivity of Al_5_Ni_2_Zr is preferable to AlNi_2_Zr. What is more, AlNi_2_Zr has a smaller electron effective mass and larger atomic non-localization than Al_5_Ni_2_Zr, similar to the bigger width of the band structure. Al_5_Ni_2_Zr with the tetragonal system is expected to be a conductor. Figure 5 presents the total density of states of Al_x_Ni_y_Zr_z_ under pressure from different pressures. It shows that the Fermi surface of AlNiZr moves higher, which is consistent with the ground energy of −8305.84, −8305.57, −8034.95, and −8034.12 ev. The Fermi surface of Al_2_NiZr_6_ moves higher, which is in agreement with the increasing ground energy of −9346.73, −9346.33, −9345.36, −9344.18, −9342.70, and −9341.07 ev. The Fermi surface of AlNi_2_Zr moves higher, which is in agreement with the increasing ground energy of −16,526.55, −16,526.17, −16,525.26, −16,524.01, −16,522.54, and −16,520.91 ev. For Al_5_Ni_2_Zr, the Fermi surface moves higher, which is in agreement with the increasing ground energy of −9154.82, −9154.37, −9153.28, −9151.83, −9154.37, and −9148.31 ev.

### 3.3. Superconducting Properties

By using the calculated elastic and electronic properties of the Al_x_Ni_y_Zr_z_, the possible superconducting properties were discussed. It can be learned from the simplified theory of superconductivity that the material needs to meet three conditions to become a superconductor. Firstly, the crystal’s atoms are lighter. Secondly, the coefficient of elasticity of the crystal is as large as possible and the crystal is tough enough. Thirdly, the material should be tantamount to the lower effective Fermi level. Al_x_Ni_y_Zr_z_ meets the first condition. What is more, Al and Zr are among the 28 superconducting elements. By observing the result of the elastic properties, Al_5_Ni_2_Zr is tougher than Al_2_NiZr_6_, AlNiZr, and AlNi_2_Zr. From the third point of view, both of them are the metal system. In conclusion, they are all liable to be used as a superconducting material. However, Al_5_Ni_2_Zr is the best of them when used as a superconducting material [26].

### 3.4. Difference Charge Density

**Figure 6:**
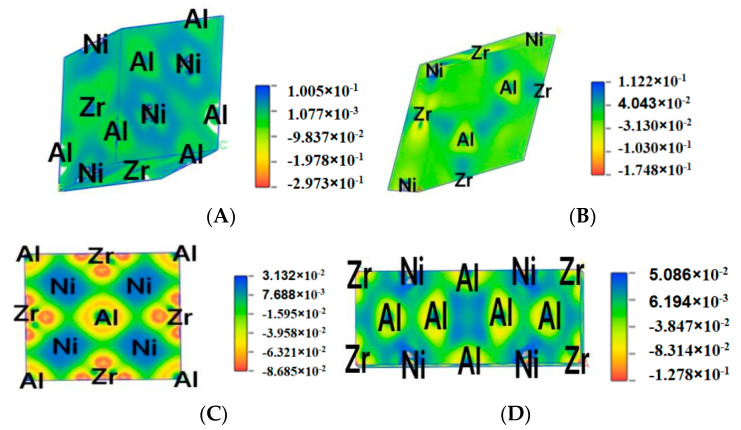
Difference charge density maps of Al_x_Ni_y_Zr_z_, (**A**) AlNiZr, (**B**) Al_2_NiZr_6_, (**C**) AlNi_2_Zr, (**D**) Al_5_Ni_2_Zr.

The difference charge density maps are shown in Figure 6, which can clearly reflect the bonding between atoms of Al_x_Ni_y_Zr_z_. By observing the plots for AlNiZr, the charge density between Ni and Ni are smaller than that between Al and Ni, Zr, and Ni, which shows that the effect between Ni and Ni is stronger. For Al_2_NiZr_6_, the charge density between Al and Zr is bigger than that of Al and Al. What is more, for AlNiZr and Al_2_NiZr_6_ with the same system, the interatomic interaction of AlNiZr is greater than that of Al_2_NiZr_6_. For AlNi_2_Zr, there is a larger charge density between Al and Ni, Zr, and Ni than Zr and Al, which means that the effect between Al and Ni, Zr, and Ni is stronger than Zr and Al. For Al_5_Ni_2_Zr, the charge density between Al and Ni is larger than that of Al and Zr, Al, and Al, which means that there is a stronger effect between Al and Ni than Al and Zr, Al, and Al. In addition, the interatomic interaction of Al_5_Ni_2_Z is greater than that of Al_2_NiZr_6_.

## 4. Conclusions

The first-principle study on elastic and electronic properties of hexagonal AlNiZr and Al_2_NiZr_6_, cubic AlNi_2_Zr, as well as tetragonal Al_5_Ni_2_Zr under pressure from 0 to 50 GPa have been calculated by using the plane-wave, ultra-soft, pseudopotentials technique within the generalized gradient approximation (GGA). The elastic constants, volume, and density of states change under pressure are analyzed. The volume decreases and the ground energy of material increases with the increase of the pressure, which is in agreement with the Fermi surface moves to be higher. The elastic constants *C_ij_*, Shear modulus *G* (GPa), Bulk modulus *B* (GPa), Poisson’s ratio *σ*, Young’s modulus *E* (GPa), and the ratio of *G/B* have been calculated under pressure from 0 to 50 GPa. Young’s modulus indicated that the relationship between the stiffness of Al_x_Ni_y_Zr_z_ is Al_5_Ni_2_Zr > AlNiZr > Al_2_NiZr_6_ > AlNi_2_Zr. The conditions are met at 30 and 50 GPa, respectively. For Poisson’s ratio, AlNiZr from 0 to 30 GPa and AlNi_2_Zr as well as Al_2_NiZr_6_ from 0 to 50 GPa are all a central force, while Al_5_Ni_2_Zr is a non-central force from 0 to 50 GPa. The *G/B* ratios for AlNiZr, AlNi_2_Zr, Al_2_NiZr_6_, and Al_5_Ni_2_Zr classify these materials as brittle under zero pressure, while with the increase in pressure the *G/B* ratios of AlNiZr, AlNi_2_Zr, Al_2_NiZr_6_, and Al_5_Ni_2_Zr all become lower, which indicate that the pressure could enhance the brittle properties of these materials.

By observing the density difference charge, the effect between Ni and Ni is stronger for AlNiZr. The effect between Al and Zr is bigger than that of Al and Al for Al_2_NiZr_6_. What is more, for AlNiZr and Al_2_NiZr_6_ with the same system, the interatomic interaction of AlNiZr is greater than that of Al_2_NiZr_6_. For AlNi_2_Zr, the effect between Al and Ni, Zr, and Ni is stronger than Zr and Al. For Al_5_Ni_2_Zr, there is a stronger effect between Al and Ni than Al and Zr, Al, and Al. In addition, the interatomic interaction of Al_5_Ni_2_Z is greater than that of Al_2_NiZr_6_.

The relationship between the electrical conductivity of Al_x_Ni_y_Zr_z_ is Al_2_NiZr_6_ > Al_5_Ni_2_Zr > AlNi_2_Zr > AlNiZr. What is more, AlNi_2_Zr has a smaller electron effective mass and larger atomic non-localization than Al_5_Ni_2_Zr. By studying the elastic and electronic properties, they all have the potential be used as a superconducting material. However, Al_5_Ni_2_Zr is the best of them when used as a superconducting material.

## Figures and Tables

**Table 1:** The elastic constants *C_ij_*, the Shear modulus *G* (GPa), Bulk modulus *B* (GPa), Poisson’s ratio *σ*, Young’s modulus *E* (GPa), for (1) AlNiZr, (2) AlNi_2_Zr, (3) Al_2_NiZr_6_, (4) Al_5_Ni_2_Zr at various pressures.

Physical Quantities		*G*				*B*				*E*				*G/B*				*σ*		
P (GPa)	1	2	3	4	1	2	3	4	1	2	3	4	1	2	3	4	1	2	3	4
0	64	45	54	87	140	123	106	107	167	120	137	205	0.46	0.37	0.51	0.81	0.30	0.34	0.28	0.18
10	77	48	65	105	184	133	142	134	203	111	170	249	0.42	0.32	0.46	0.78	0.32	0.36	0.29	0.19
20	88	56	74	124	220	240	165	191	233	155	192	306	0.40	0.23	0.45	0.65	0.32	0.39	0.31	0.23
30	98	65	80	143	250	274	213	212	261	181	213	350	0.39	0.24	0.38	0.68	0.33	0.39	0.33	0.22
40	…	64	83	155	…	313	240	270	…	179	223	392	…	0.20	0.35	0.57	…	0.41	0.35	0.26
50	…	65	88	168	…	343	262	303	…	184	236	426	…	0.19	0.30	0.55	…	0.41	0.35	0.27
